# Disease Modeling Using 3D Organoids Derived from Human Induced Pluripotent Stem Cells

**DOI:** 10.3390/ijms19040936

**Published:** 2018-03-21

**Authors:** Beatrice Xuan Ho, Nicole Min Qian Pek, Boon-Seng Soh

**Affiliations:** 1 Disease Modeling and Therapeutics Laboratory, A*STAR Institute of Molecular and Cell Biology, 61 Biopolis Drive Proteos, Singapore 138673, Singapore; xbho@imcb.a-star.edu.sg (B.X.H.); mqnpek@imcb.a-star.edu.sg (N.M.Q.P.); 2 Department of Biological Sciences, National University of Singapore, Singapore 117543, Singapore

**Keywords:** 3D organoids, disease modeling, human induced pluripotent stem cells, microenvironment, neurodevelopmental disorders, drug screening, infectious diseases, hereditary diseases, genome editing, CRISPR/Cas-9

## Abstract

The rising interest in human induced pluripotent stem cell (hiPSC)-derived organoid culture has stemmed from the manipulation of various combinations of directed multi-lineage differentiation and morphogenetic processes that mimic organogenesis. Organoids are three-dimensional (3D) structures that are comprised of multiple cell types, self-organized to recapitulate embryonic and tissue development in vitro. This model has been shown to be superior to conventional two-dimensional (2D) cell culture methods in mirroring functionality, architecture, and geometric features of tissues seen in vivo. This review serves to highlight recent advances in the 3D organoid technology for use in modeling complex hereditary diseases, cancer, host–microbe interactions, and possible use in translational and personalized medicine where organoid cultures were used to uncover diagnostic biomarkers for early disease detection via high throughput pharmaceutical screening. In addition, this review also aims to discuss the advantages and shortcomings of utilizing organoids in disease modeling. In summary, studying human diseases using hiPSC-derived organoids may better illustrate the processes involved due to similarities in the architecture and microenvironment present in an organoid, which also allows drug responses to be properly recapitulated in vitro.

## 1. Introduction

The global rise in the number of patients suffering from chronic and/or degenerative diseases has not only led to a tremendous increase in the demand for an unlimited source of transplantable tissues, but also sustainable human disease models that will allow for in-depth investigations into disease pathogenesis. Harvesting cadaveric donor tissues, which in itself is a limited source, for both transplantation and disease modeling is highly inefficient and logistically challenging, often resulting in low yield of functional cells/tissues. This then has fueled the advancement of cellular regenerative therapy of which the primary focus lies in tapping into the pluripotency of human embryonic stem cells (hESCs) (and even extending towards the use of organ-restricted adult stem cells) to generate large quantities of transplantable somatic cell types in vitro in the presence of defined culture conditions; a process also known as directed differentiation. Since hESCs are proliferative and capable of self-renewal, they can be used to generate transplantable cells, as well as different cell types that can be manipulated to model human diseases.

Ethical and immuno-compatibility issues faced due to the use of hESCs and its derivatives for clinical transplantation have been overcome by the advent of the human induced pluripotent stem cell (hiPSC) technology where patient-specific somatic cells (e.g., skin fibroblasts and hematopoietic cells) can be directly reprogrammed by defined factors to induce pluripotency [1]. These hiPSCs displayed similarities in morphology, proliferation, feeder dependence, surface markers, gene expression, promoter activities, in vitro differentiation potential, and teratoma formation characteristics to hESCs. This is coupled with the fact that hiPSCs are patient-specific and would have virtually no risk of immune rejection when hiPSC-derived cells are transplanted back into the patient. In addition, the patient-specific nature of hiPSCs has provided researchers with an indispensable tool for modeling a diverse range of diseases in vitro. The generation of patient-specific iPSCs through cellular reprogramming [2] has shown tremendous success in recapitulating disease manifestations that are very close to clinical phenotypes reported in patients [3]. The hiPSC technology has presented an unprecedented opportunity to model diseases associated with specific cell types/ tissues, especially those that are highly invasive and risky to obtain directly from living patients, such as neurons, cardiac and pancreatic cell types, etc.

Using hiPSCs to model diseases has provided an opportunity to bridge the gap between fundamental genetic studies and disease pathogenesis. Researchers can now readily generate hiPSCs from patients to launch in-depth investigations into the underlying disease mechanisms of complex diseases and even study developmental defects by recreating embryonic development through directed differentiation of those hiPSCs; such investigations were virtually impossible prior to the availability of the hiPSC technology.

Recent advancements in genome editing, such as clustered regularly interspaced short palindromic repeats (CRISPR)/CRISPR-associated system (CRISPR-Cas9) genome engineering tool, has enabled researchers to efficiently manipulate genomic sequences in hESCs and hiPSCs [4]. Several authors have highlighted the applications of patient-derived iPSCs to modeling diseases through the ability to repair putative genetic mutations in those hiPSCs, or introduce genetic mutations into healthy wild-type cell lines in order to better establish the relationship between the genetic mutations and perturbations in cellular functionalities [5,6,7,8,9,10]. Ultimately, knowledge gathered from these studies will allow for the development of cell-based platforms of varying scales for pre-clinical testing, such as drug screening and therapeutic optimization.

Robust directed differentiation of hESCs and hiPSCs into somatic cell types in 2D monolayer cell culture systems have advanced disease-modeling efforts over the past decade. Many researchers have been successful in gathering novel and crucial insights into complex diseases. However, the field has grown to realize that these conventional 2D models, such as cells obtained from monolayer differentiation methods and homogenous primary/patient cell line cultures, are still relatively limited in providing a comprehensive understanding of complicated processes such as embryonic development, cellular differentiation, tissue regeneration, and disease development [11]. Current 2D models for studying neurodegenerative disorders, for instance have not been successful in accurately recapitulating the spatial organization of neural tissues, cell–cell adhesion, and cell–extracellular matrix interactions in vitro. The need for a more accurate model system has pushed the field towards developing 3D neural structures “in a dish” [12]. These structures were then coined an “organoid”, which has today been adapted to describe a spherical (at times taking on an irregular shape) 3D mass in culture obtained from differentiating human pluripotent stem cells (hPSCs) and/or mesenchymal stem cells (MSCs) into multiple cell types that collectively exhibit tissue organization, cellular compartmentalization, and organ-like functions [13]. Essentially, 3D organoid models are cultures of aggregate-like masses that arise from a single-lineage progenitor as opposed to its 2D predecessors that take the form of single cell-type monolayer culture. In addition, a 3D organoid is also different from 3D engineered tissues, where the latter is usually formed from an assembly of different cell types. A comparison between 2D and 3D organoid culture systems are provided in Table 1.

Whilst the 3D organoid technology is still in its infancy, it has prevailed to be superior as compared to its traditional 2D counterparts. The organoids that can be obtained from well-established differentiation protocols have the capacity to self-organize and generate sophisticated 3D structures that accurately mimic the morphology of organs [14,15,16]. In contrast to 2D monotypic cellular models, 3D organoids are able to undergo multi-lineage differentiation to form a heterogeneous population of cells that self-organize into an elaborate tissue-like architecture [14,17]. Cellular migration and segregation, as well as spatially-restricted lineage commitment, marks the foundation for tissue self-organization during organogenesis [18]. The organoid culture system allows for these fundamental processes to occur in vitro, thus rendering them successful in allowing for cellular organization to happen and, as a result, establishing a physiologically more relevant microenvironment as compared to the 2D cultures. Moreover, as the field aims to transcend beyond the use of animal models, the 3D organoids obtained from hPSCs are advantageous in modeling human diseases and allow for the manipulation of niche components, such as signaling pathways and transcriptional and translational regulators, due to its in vitro nature.

Unsurprisingly, this has spurred recent reports of various differentiation protocols to obtain a myriad of organoid types (modeling different tissues) in vitro, such as intestinal, kidney, brain, retinal, pancreatic, and liver organoids. While organoids have been derived from several tissues or organs, cardiac organoids have yet to be reported, although several groups have reported generation of 3D cardiac tissues in vitro [19]. By directing hPSCs to differentiate to form different germ layers—endoderm, mesoderm, and ectoderm—progenitors arising from different lineages will then be aggregated together and further differentiated into the respective cell types/tissues of interest. With that, many complex diseases associated with different organs can now be modeled using these various forms of organoids, as summarized in Table 2.

In this review, we will discuss how hiPSCs can differentiate into different types of organoids and how genetic manipulation of genes using advanced genome editing tools can be used in understanding normal embryonic and disease development and, eventually, provide avenues for disease modeling in vitro. The knowledge gathered from such disease models can subsequently be translated into therapeutic options. Herein, we have also highlighted some strengths of modeling human diseases in 3D and address the current limitations that are plaguing this culture system. Perhaps while 3D organoid technologies are still developing and improving, utilizing this modern methodology to model diseases in tandem with the use of more traditional approaches such as animal models will be a powerful and holistic approach in modeling numerous types of complex human diseases.

## 2. Recognizing Limitations of both 2D Monolayer and 3D Organoid Models

### 2.1. Limitations of Conventional 2D Monolayer Models

#### 2.1.1. Non-Natural and Static

The 2D cell culture models still predominantly used place cells in a non-natural environment with cells growing in a homogeneous monolayer and adhering themselves on a bed of artificial matrix that attempts to mimic the extracellular matrix (ECM). Though such an experimental setup proves to be both simple and efficient, it does not provide cells with the natural biophysical and biochemical environmental cues that have a significant impact in cellular activities and functionalities [51]. The monotypic nature of many 2D models also does not recapitulate the complexity of the microenvironment adapted by the heterogeneous population of cells in vivo. In the field of disease modeling where the aim is to ultimately recapture and understand the pathophysiological mechanisms of diseases in vitro, this raises questions about the suitability of 2D cultures in providing the precision that disease modeling demands.

#### 2.1.2. Microtopography and Types of Cell Culture Surfaces

Many recent studies have shown that the attributions of the surfaces that cells grow on in 2D models influence the cellular and molecular characteristics of the cells, such as cell development, proliferation, differentiation, apoptosis, and cell migratory behaviors [52]. The microtopography and the nature of the substrates of cell culture surfaces have a direct impact on the self-renewal and pluripotency-maintenance properties when culturing hPSCs, as well as the downstream differentiation potential of these stem cells [53,54]. Microtopography and substrate stiffness of a cell culture surface have been shown to affect cell migration, which could also influence cellular differentiation and development. At present, a myriad of matrices is available to improve stem cell morphology before and during differentiation. For other 2D models involving primary cell cultures, patient-obtained cells, or transformed cells, considerations on which substrate to use is important as it has an impact on various crucial phenotypes, such as cellular organization and functionalities of cells in the case of neurons, osteoblasts, and transformed cells such as prostate cancer cells and cardiomyocytes [55,56,57,58]. Therefore, it is crucial to consider that given the static nature of 2D cellular models and how cells are grown on different types of substrates, these culture systems are highly artificial and do not mimic the natural development of cells and tissues, which involve complex and dynamic cell migratory behavior and cell-to-ECM interactions. Cells in 2D models have also been shown to exhibit differing gene and protein expression profiles compared to 3D models [59]. This potentially limits 2D models in providing thorough and biologically relevant information about disease mechanisms, which could ultimately hinder the advancements of therapeutics for those diseases.

### 2.2. Limitations of Organoid Models

#### 2.2.1. Reproducibility

Current organoid systems have reproducibility concerns as there is little to no control over how cells self-organize into the organoids. Therefore, these protocols are unable to guarantee exact replications of organoid spheres of the same dimensions (i.e., size and shape), cellular composition, phenotypic and molecular characteristics [60]. The autonomous potential of organoids to arrange and develop into tissue-like structures is a remarkable strength but also a weakness because progressing an organoid system into clinical and pharmaceutical settings requires the production of consistent organoids for therapeutic quality control and safety. Although recent improvements have been made in allowing for standardization and better control of deriving organoids such as cortical organoids [61], the stem cell niche was proposed to be a major aspect in which researchers can manipulate to improve the reproducibility of the organoids obtained [62]. However, the current standards are still miles from good manufacturing practices grade.

#### 2.2.2. Vascularization

Researchers have begun to recognize the importance of generating vascularized organoids. Vascularization will allow for oxygen and nutrients to be supplied throughout the mass, encouraging better development of cells into tissue-like structures and for the cells that are within the mass to be able to survive and function as well as cells on the periphery [62]. From a therapeutic perspective, a vascularized organoid will be able to capture the drug uptake, circulation, and metabolism that occur in the body. From a transplantation perspective, a vascularized organoid could be able to be grafted into patients as it could be integrated with the patient’s vascular network. 

However, while promoting neo-vascularization of organoids is no simple task, great strides have been made in devising methods to vascularize 3D cellular models. Vascularized 3D cancer models have been generated to study tumorigenesis and angiogenesis [63]. Vascularized 3D adipose tissue was generated in an attempt to improve the generation of soft tissues, such as adipose tissue, for regenerative therapy [64]. Recently, a bio-printed 3D vascularized tissue model was published that allows a more accurate model for drug administration and toxicity analysis [65]. However, current techniques have yet to produce organoids that comprise an extensive vascular network comprising of robust layers of endothelial and vascular smooth muscle cells (VSMCs) that shows functional phenotypes such as endothelium permeability or VSMC contraction.

#### 2.2.3. Blood Perfusion and Inflammation

While existing 3D organoid protocols have revolutionized our approach to disease modeling and brought much success in recreating the intricate interactions with various cell types in a spherical micro-environment, these models are still unable to recapture the complex and dynamic inflammation niche that occurs in vivo and that involves a multitude of cell types (endothelial cells, monocytes, macrophages, leukocytes) and cellular processes (leukocyte-endothelial cell adhesion, leukocyte extravasation and transmigration [66], and monocyte to macrophage differentiation). Blood is undoubtedly crucial for the body’s inflammatory response by serving as the carrier medium for immune cells. These immune cells eventually home to sites of injury through chemo-attraction and interact with blood vessel walls (through adhesion molecules presented by endothelial cells) before extravasating to inflamed loci. Therefore, in order to mimic inflammation in 3D in vitro models, vascularization has to first be established, blood perfusion has to occur, and immune cell types must be present. A 3D model that can successfully incorporate such an inflammation niche will be an extremely powerful tool for studying atherosclerotic vascular diseases, inflammatory skin diseases, interstitial nephritis, and even inflammatory bowel disease.

Several 3D skin models (non-organoid-based) published within the last decade have attempted to model inflammatory skin diseases, which are the most common dermatological pathologies. These models have attempted to address the perplexing task of modeling inflammation through the utilization of various techniques that are technically demanding. One of the earlier models made use of a layering technique to create a multi-layered 3D skin structure supported by collagen matrices and exposed to an air–liquid culturing interface to mimic actual skin (also known to the field as “raft cultures”) [67]. The authors showed that this model is able to respond to irradiation insults and produce a pro-inflammatory response. Building on this “raft culture” technique, Linde et al. reported on co-culturing macrophages with the 3D skin structure in their model system [68]. Since macrophages are able to interact indirectly with the skin structure, this system allows studies to be performed on the inflammatory dynamics between macrophages and skin. The authors were able to illustrate that the 3D skin structure was able to perform its function as a skin barrier, thereby ameliorating immune response of macrophages upon inflammatory stimuli. Although the authors only co-cultured the 3D skin structure with macrophages, there is the potential for this system to include multiple immune cell types, possibly bringing 3D in vitro models one-step closer to representing the bona fide inflammation niche. However, it is still paramount to recognize that these ‘raft culture’ methods are static and do not encompass the perfusion of blood. Blood perfusion, in the context of inflammation, plays critical roles in vessel hyperpermeability and vasodilation, which occurs during the onset of acute inflammation. Therefore, “raft cultures” still do not recapitulate the complexity of an actual immune response.

One of the earlier works on engineering blood perfusion in organoids was published by Sefton in 2006 where they allowed for modular assembly of endothelial cells with “solidified cells (in collagen gel rods)” that were assembled into a larger tube where fluid or whole blood could pass through [69]. However, the authors referred to their setup as a “vascularized organoid”, which in accordance to the above-mentioned definition is inaccurate as the cells were embedded in rods and not allowed to self-organize into a mass. More recently, Wufuer et al. created a skin-on-chip model that recaptured the intimate interactions between epithelial cells, fibroblasts, and endothelial cells through the incorporations of microfluidics technology. This 3D-based platform allows readouts on skin permeability in the presence of fluid profusion [70].

#### 2.2.4. Humanized Mice Models Provide a Systemic Environment for Disease Modeling

Murine models have been extremely helpful in disease modeling prior to the advent of the organoid technology. Immunodeficient mice such as the nude mouse and severe combined immunodeficiency (SCID) mouse have been commonly used as recipients of human cells or tissues as they accept foreign cells relatively easily due to a lack of host immunity. In contrast, humanized mice are rodents that have been removed from an innate immune system and completely re-populated with human immune cells by human hematopoietic stem cell transplantation [71]. Such humanized mouse models are particularly useful to model disease pathology and to allow for the assessment of potential therapeutic candidates in an in vivo setting that is relevant to human physiology. They provide a systemic environment to holistically study disease pathology due to the presence of an intact immune system and blood circulation, which are essentially impossible in 2D models. Many studies have been successful in providing new insight into disease pathogenesis and immunology-disease interactome using humanized rodents to model human diseases [72]. However, researchers have begun to realize and report on the distinct differences between rodent and human immunology [73], thus raising questions on the relevance of using mouse models to study human diseases. Nonetheless, 3D organoids serve to circumvent the challenges faced by 2D culture and animal disease models. However, such modeling of human diseases in 3D organoids may still need to be validated in vivo eventually using a humanized mouse model.

## 3. Therapeutic Applications of 3D Organoids

**Figure 1:**
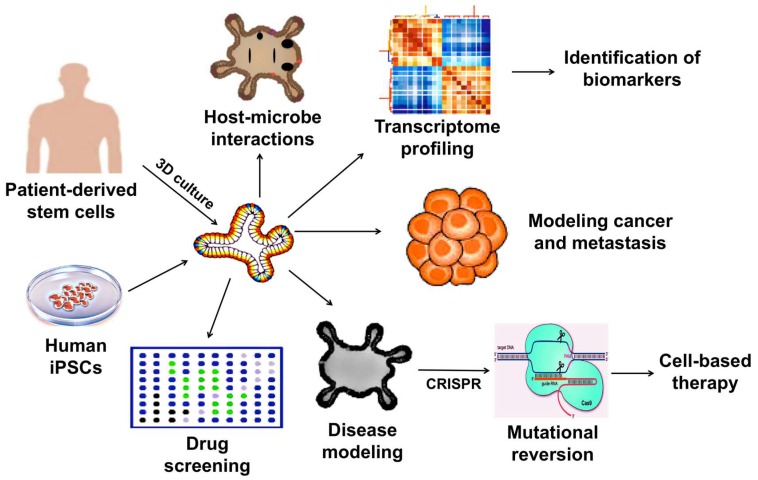
Applications of 3D organoids in therapeutic and pharmaceutical testing.

Considering the limitations together, the 2D culture systems may no longer be useful in human disease modeling. In addition, the use of animals for disease modeling, drug testing, and therapeutic development are not only costly and time consuming but may not faithfully represent biological responses in humans due to species differences. Hence, whenever possible, it is a strategic choice to use organoid-based methods to investigate regulatory and pathological mechanisms at a molecular level. The wide-array of biomedical applications of which 3D organoids can be utilized is illustrated in Figure 1.

### 3.1. Development of Drug Screening Platforms

#### 3.1.1. Cerebral Organoid Platforms for Drug Screening

Organoid culture is a powerful tool for modeling neurodevelopmental disorders, such as primary microencephaly, and can be employed as a drug-screening platform. Due to an increasing incidence of infants born with severe microencephaly, many studies have presented evidence of prior/premature exposure to Zika virus [3,14,74]. In several studies, hESC-derived cerebral organoids have revealed that Zika virus (MR766) depleted neural progenitors during the early stages of brain development (first trimester) [11,12,74].

3D models of neural stem cells (NSCs) in the form of neurospheres and brain organoids were used to explore implications of Zika virus (ZIKV) infection during neurogenesis and growth [12]. Interestingly, these models showed that ZIKV infection led to morphological defects and impeded growth of neurospheres. Further comparisons between mock- and ZIKV-infected NSCs revealed pyknosis of the nucleus, mitochondrial swelling, smooth membrane structures, and viral envelopes, similar to that with the dengue virus. These pathological signatures were successfully revealed and modeled in vitro. The model was reported to possess gene expression profiles that indicate ZIKV infection induces death in human neurospheres during fetal developmental, which could lead to severe tissue damage [12,75]. The organoid models also showed upregulation of Toll-like receptor 3 (*TLR3*) gene upon ZIKV infection, which was associated with perturbed cell fate differentiation and reduced organoid volume, both of which physiologically mimic clinical manifestations of microencephaly [11].

Cerebral organoids have been able to express heterogeneity and regionalization of several regions of the brain, including the cerebral cortex, ventral forebrain, midbrain–hindbrain boundary, and hippocampus [75]. Upon successful neural induction, upregulation of neural identity markers *SOX1* and *PAX6* were observed [17]. Cerebral organoids have recapitulated distinct development characteristics of specified brain regions; forebrain markers (*BF1* and *SIX3*) remained highly expressed whereas hindbrain markers (*KROX20* and *ISL1*) decreased [17]. Lancaster et al. utilized patient-derived iPSCs and shRNA with CDK5RAP2 mutation in these organoids to model microencephaly [17]. More recently, Li and colleagues demonstrated cerebral organoids obtained from iPSCs that were generated from a patient with abnormal spindle-like primary microcephaly (ASPM) were capable of recapitulating neurogenesis defects in the disease [20]. The cerebral organoids consisted of organized progenitor zones. The sub-ventricular zone (SVZ) was split by an inner fiber layer (IFL) into the inner SVZ and outer SVZ. Additionally, cerebral cortical neurons displayed complex branching and growth behavior with long-range axon projections resembling axonal bundling [17]. Neurons within cerebral organoids also revealed spontaneous Ca^2+^ oscillations in single cells. Treatment of these cells obtained from the organoid showed that tetrodotoxin (TTX)-induced action potential blockade dampened calcium surges, thus indicating calcium spikes were dependent on neuronal activity. Finally, the authors validated the functionality of glutamatergic receptor activity of the developing neural tissue [17]. Cerebral organoids mimic the human cerebral cortical organization and function illustrating its superiority in 3D modeling of aberrant neurodevelopmental processes.

Collectively, brain region-specific organoids can model neurodevelopmental disorders and possible compound testing for ZIKV antiviral drugs as demonstrated by Zhou et al. Their group identified two potential drug compounds, hippeastrine hydrobromide (HH) and amodiaquine dihydrochloride dihydrate (AQ), which can inhibit ZIKV infection in human pluripotent stem cell-derived cortical neural progenitors cells (hNPCs), as well as rescue the effects of ZIKV-induced growth and differentiation defects in hNPCs and human fetal-like forebrain organoids [76].

#### 3.1.2. Modeling Hepatic and Biliary Development for Drug Screening

Organoids derived from adult liver and pancreas also provide a platform for regenerative medicine and disease modeling [77]. Takebe et al. demonstrated that co-culture of hiPSC-hepatic endoderm with human umbilical vein endothelial cells and mesenchymal stem cells (MSCs) resulted in the self-organization of hiPSC liver buds [78]. The authors hypothesized that the presence of MSCs formed a mechanical support to initiate condensation within the heterotypic cell mixture, thereby promoting the formation of 3D vascularized and functional liver buds [79]. Although human adult hepatocytes remain the gold standard for in vitro toxicology tests, their low replicative capacity, in addition to the poor quality arising from rapid dedifferentiation occurring during culture, have driven researchers to consider ESCs and iPSCs-derived organoid culture as a good source of hepatocyte-like cells [80]. Since liver organoids have hepatocellular differentiation potential, they can provide an unlimited source of hepatocytes for applications in drug screening and for the design of personalized treatments.

### 3.2. Modeling Infectious Diseases

Induced human intestinal organoids (iHIOs) can be generated to mimic the human intestine through exposure to a series of growth factors to mimic embryonic intestinal development [69]; Activin-A induces definitive endoderm (DE) formation, FGF/WNT induces posterior endoderm patterning, hindgut specification, and morphogenesis. FGF/WNT also promotes a pro-intestinal culture system to encourage intestinal growth, morphogenesis, and cytodifferentiation. After 28 days in culture, iHIOs were found to express intestinal stem cell markers and consist of polarized, columnar epithelium patterned into a villus-like structure that collectively exhibited similar morphology to human intestinal epithelium [81]. Furthermore, iHIOs displayed physiological characteristics to undergo maturation in vitro and acquire both absorptive and secretory functions [81].

The ability to generate iHIOs greatly opens the field of translational cell-based therapy for diseases such as necrotizing enterocolitis, inflammatory bowel disease, and short gut syndromes [81]. Earlier studies by Finkbeiner et al. showed that the iHIO model has the ability to act as a host for replication of human patient-obtained rotavirus strains in vitro [30]. These organoids displayed capabilities to produce infectious rotaviral particles [31], thereby making them suitable models for studying intestinal viral infection.

The induced human intestinal organoids can also model other host–microbe interactions with pathogens such as *Helicobacter pylori* and *Salmonella enterica*, where anti-viral/bacterial therapy testing can be done on such models [82]. Recently, a study by Amieva et al. highlighted how human gastric organoids present a potential model for linking colonization of *Helicobacter pylori* in the human stomach and its progression to stomach cancer [37].

### 3.3. Modeling Cancer

New frontiers of modeling cancer in vitro have included the use of patient cell-derived tumor organoids. Tumor organoids can be efficiently generated from either circulating tumorigenic cells, cancer cell lines, or cells extracted from tumors, and are highly expandable [83,84], thus providing researchers with adequate material for modeling specific forms or even rare types of cancers and for large-scale drug development and screening. The spatial and cellular architectural aspects of the organoid cell culture prove to be better than traditional cancer models such as cell lines or patient-derived xenograft models. Tumor organoids adequately represent tumor heterogeneity seen in patient tumors and can be used to anticipate in vivo drug sensitivity and resistance [44]. A review addressed by Edmondson et al. discussed the use of a 3D biosensor-based assay for multiple analysis of various anticancer drugs on a variety of tumor organoids [59].

#### 3.3.1. Prostate Cancer

Earlier prostate cancer organoids (PCOs) were generated from patient biopsy samples and circulating metastatic cancer cells [43]. These organoids were able to mimic in vivo tumor histology and molecular profiles similar to the patients. Recurrent genomic mutations prevalent in metastatic prostate cancer subtypes, including *PTEN* loss, *TMPRSS2-ERG* interstitial deletion, *SPOP* mutation, *SPINK1* overexpression, *FOXA1* mutation, and *CHD1* loss, were also observed in the organoid models [44]. This implies that PCOs can recapitulate the mutational landscape that is clinically defined and is a suitable model for better understanding the complex and unknown mechanisms involved in disease progression to the more severe metastatic castrate-resistant prostate cancer (CRPC). As demonstrated recently by Saeed et al., PCOs can be used in high-throughput comprehensive drug response studies and the results of the test highlighted known and novel drug sensitivities [85].

#### 3.3.2. Colorectal Cancer

Patient-derived intestinal stem cell-derived tumor organoids marked by *LGR5* recapitulated several properties of the original tumor architecture, cell composition, and self-renewing capabilities. Tumor organoids generated from healthy epithelium and tumor-derived organoid cultures provide a comparative model for investigating the causal role of genetic mutations leading to colorectal cancer pathogenesis. While wild-type intestinal organoids expressed goblet cell genes such as *MUC1, MUC4*, and *CA2* (colonocyte marker), tumorigenic organoids were enriched with cancer-associated genes such as *PROX1*, *BAMBI*, *PTCH1* and *APCDD1* [36]. Additionally, these colorectal tumor organoids composed of a heterogeneous population of cells, which provides a more holistic microenvironment that could account for drug resistance and metastatic potential of the tumorigenic cells. Hence, applications of the tumor organoid technology can be highly effective in revealing clinically relevant biomarkers that underpin drug sensitivity and exploits the relevance of tumor heterogeneity to personalized medicine [36].

In addition, Drost et al. recently demonstrated that CRISPR/Cas9-mediated genome editing was capable of generating human intestinal cancer stem cells by inducing four most commonly mutated colorectal cancer genes (*APC*, *P53*, *KRAS*, *SMAD4*) [35]. Mutant intestinal organoids injected into immuno-deficient mice displayed growth of intestinal tumors possessing invasive carcinoma in vivo. Similarly, in vitro triple and quadruple mutant human intestinal organoids displayed highly proliferative characteristics, whereas only quadruple mutant-derived organoids resulted in solid tumor masses [35].

#### 3.3.3. Ovarian Cancer

Fallopian tube epithelium (FTE) has been described as one of the sites of origin of high-grade serous ovarian cancer (HGSOC). Traditional 2D FTE culture systems have failed to recapitulate the architecture and geometrical features of tissues in vitro, as well as gradients of nutrients, oxygen, and carbon dioxide that are the fundamental tissue environments in vivo. On the other hand, 3D organoid-derived FTE reported by Lawrenson et al. demonstrated polarized columnar epithelium containing the desired cell types and convoluted luminal architecture, which closely represents the structure and organization of the fallopian tube in vitro [42,86]. The differentiated FTE presents biological relevance as it recapitulates physiologically relevant aspects of disease progression in the presence of secretory and ciliated epithelial cell components in vitro.

The derivation of fallopian tube organoids (FTOs) from adult stem cells came only recently when Kessler et al. described the formation of FTOs using stem cell-like cells obtained from gynecological FTE tissue samples [86]. The FTOs were more elaborate and were observed to have a mucosal fold architecture that is seen in human fallopian tube tissues. They were also shown to be responsive to a hormonally-active environment, which is a crucial aspect in the carcinogenesis of ovarian cancer. Therefore, FTOs have the capacity to be a suitable and expandable model for studying various forms of ovarian cancer.

### 3.4. Modeling Hereditary Diseases

#### 3.4.1. Cortical Organoids

Autism spectrum disorder (ASD) is a neurodevelopmental disorder that can be modeled using neural cortical organoids generated from iPSCs obtained from individuals with severe idiopathic ASD. These cortical organoids exhibited a decreased cell cycle duration, indicative of perturbed cell cycle potential, and showed an overproduction of GABAergic inhibitory neurons, providing critical insight into the pathogenesis of ASD that was previously unclear. The success of cortical organoids in modeling ASD is largely due to the ability to model embryonic telecephalic development seen in the third trimester of human development, as well as recapturing the regulatory networks of GABAergic neuron production. The authors identified gene expression of *FOXG1* could potentially be used as a biomarker of severe ASD [21]. Dysregulation of *FOXG1* gene predominant in these cortical organoids provides an understanding of the alterations in the dynamics of brain growth and differentiated neurons. A separate study by Mariani et al. reported that organoids displayed organized layers of radial glia, intermediate progenitors, and neurons, and that several pathological features in these organoids were observed, which correlates to earlier studies suggesting an increased number of neurons [87] and an increased number of cortical mini-columns [88] and synapses [89] in ASD individuals. Collectively, this model provides a framework for functional studies, such as whole-cell patch-clamping from individual neuronal cells that are obtained from dissociating the organoids, thus allowing for electrical excitation and action potential read-outs of ASD organoids against familial controls [21].

#### 3.4.2. Intestinal Organoids

Intestinal organoids are not only capable of modeling host–microbe interactions (as mentioned in Section 3.2) but also other diseases such as cystic fibrosis (CF). Schwank et al. have published studies showing patient-derived primary intestinal stem cells can be used to generate intestinal organoids that can be a good model of CF [34]. Cystic fibrosis is an autosomal recessive disease caused by the cystic fibrosis transmembrane-conductor regulator (*CFTR*) gene mutation. The *CFTR* gene encodes for the ATP-binding cassette transporter and *CFTR* mutations affect the liver, lung, pancreas, and intestine [26]. CFTR protein has a functional role in chloride channels and controls ions and water secretion and absorption in epithelial tissues. Correction of the *CFTR* mutation using CRISPR/Cas9-mediated gene editing in primary adult intestinal stem cells fully restored the functional phenotype in intestinal organoids in culture [5,34].

#### 3.4.3. Hepatic Organoids

Whilst hepatic organoids (HOs) may be useful in drug screening and toxicology studies (as mentioned in Section 3.2), they can also be used to model other forms of diseases such as Alagille syndrome (ALGS). ALGS is an autosomal dominant genetic disorder predominantly (94%) caused by mutations in the *JAG1* gene that encodes for NOTCH ligand JAG1 [24]. Clinical pathology of ALGS consists of liver damage caused by the abnormal formation of bile ducts, resulting in abnormal development of biliary processes [24]. Nicholas et al. have developed a technique of culturing hiPSC-derived HOs in vitro to form endoderm spheres in the presence of growth factors and chemicals according to a modified protocol as mentioned [90]. More recently, HO formation was further induced by matrigel encapsulation and the addition of FGF10, known to promote differentiation of foregut endoderm into hepatic and gallbladder cells during organogenesis [24]. Patient-specific iPSC-derived HOs recapitulated clinical phenotypes of ALGS, which include the absence of apparent tubular structures and somewhat fewer albumin-positive hepatocytes than control HOs. Additionally, ALGS iPSCs displayed significantly attenuated organoid formation efficiency with over 90% of vesicular structures in ALGS cultures, whereas over 50% of structures formed in wild-type HO cultures were intact organoids [24]. ALGS organoids also showed markedly reduced expression of mRNA for NOTCH 2 and bile duct mRNA differentiation markers (*CK19, CK7, GGT*, and *CFTR*) [24].

As a proof of concept, the authors generated an isogenic control through the correction of ALGS1 mutation (ALGS1^rev^) and introduced two ALGS mutations (C1^mu^ and C2^mu^) in iPSCs generated from two different control individuals [24]. The results obtained showed that HOs generated from iPSCs with ALGS^rev^ rescued the disease phenotype and recovered their capacity to form well-organized structures with bile ducts [24]. On the contrary, C1^mu^ and C2^mu^ formed less compact and well-organized bile duct structures compared to wild-type HOs [24]. However, in the reversion of the ALGS genetic mutation to wild-type, organoids displayed a marked increase in differentiated hepatocytes and cholangiocytes. 

In addition, a recent study by Kruitwagen et al. reported the generation of a long-term feline HO culture to model hepatic steatosis using adult liver stem cells. The HOs were demonstrated to be successful in exhibiting lipid accumulation and disrupted β-oxidation metabolism [91]. Evidently, this study showed that HOs could also be used to model liver metabolic diseases with no clear underlying genetic aberrations. 

## 4. Conclusions

Organoid technology holds great potential in disease modeling and has provided researchers with new hopes of generating animal-free, scalable, and more complex human disease models that can more accurately portray clinical phenotypes. While tremendous progress has been made over the past decade on organoid technology, and many studies have provided convincing evidence supporting the use of organoids in human disease modeling, there remain several limitations in the inability for certain tissue stem cells (e.g., cardiac stem cell) to form organoids in vitro. As such, while researchers are still juggling between the strengths and weaknesses of 2D and 3D cellular models in disease modeling (as summarized in Table 1) in order to determine the best strategy to approach their studies, more time should be given to allow the development of the organoid technology for modeling human diseases. Perhaps the best advice at present is to rely on a combination of 2D and 3D organoid models (if available), coupled with humanized animal models, to provide a comprehensive and reliable understanding of the molecular mechanisms involved in human diseases.

## Figures and Tables

**Table 1:** A comparison between 2D and 3D organoid model systems.

Technical and Experimental Considerations	2D Model	3D Organoid Model
Cost	Low	Moderate to high
Ease of setup	Easy to moderately challenging	Very challenging
Time required	Low	Moderate to high
Cell–ECM interactions	Artificial environment	Mimics natural environment
Reproducibility	High	Low
Vascularization	No	Yes
Blood perfusion	No	Highly possible
Inflammation	Co-culturing techniques allow for a simple way to model inflammation	More improved technologies allow for modeling the complexity of inflammation

**Table 2:** Recently established disease models involving human induced pluripotent stem cell (hiPSC)-derived organoid culture systems.

Tissue/Organ	Disease Modeled	References
**Brain**	Zika virus and congenital brain malformations	Kelava et al., 2016 [3]; Dang et al., 2016 [11]; Garcez et al., 2016 [12]; Cugola et al., 2016 [14]
	Primary microencephaly	Kelava et al., 2016 [3]; Dang et al., 2016 [11]; Lancaster et al., 2013 [17]; Li et al., 2017 [20]
	Autism/macrocephaly	Mariani et al., 2015 [21]
	Alzheimer’s disease	Raja et al., 2016 [22]
	Parkinson’s disease	Monzel et al., 2017 [23]
**Liver**	Alagille syndrome A1AT deficiency Cystic fibrosis	Guan et al., 2017 [24]; Gomez et al., 2016 [25]
**Pancreas**	Cystic fibrosis	Hohwieler et al., 2017 [26]
	Pancreatic ductal adenocarcinoma	Huang et al., 2015 [27]; Baker et al., 2016 [28]
	Diabetes mellitus	Kim et al., 2016 [29]
**Intestinal**	Host–microbe interactions e.g., human norovirus	Finkbeiner et al., 2012 [30]; Yin et al., 2015 [31]; Ettayebi et al., 2016 [32]
	Cystic fibrosis (CF)	Dekkers et al., 2013 [33]; Schwank et al., 2013 [34]
	Colorectal cancer	Drost et al., 2015 [35]; van de Wetering et al., 2015 [36]
	Host–microbial interactions (e.g., *Helicobacter pylori*)	Finkbeiner et al., 2012 [30]; Huang et al., 2015 [27]; Amieva et al., 2016 [37]; Boj et al., 2017 [38]
**Stomach**	Cancer	Takasato et al., 2015 [39]
**Kidney**	Polycystic kidney disease	Freedman et al., 2015 [40]
	Ovarian cancer	Yucer et al., 2017 [41]; Lawrenson et al., 2013 [42]
**Urological**	Prostate cancer	Gao et al., 2014 [43]; Gao et al., 2015 [44]
**Lung**	Fibrotic lung disease	Dye et al., 2015 [45]; Barkauskas et al., 2017 [46]; Chen et al., 2017 [47]
**Retinal**	Leber congenital amaurosis (LCA), Retinitis pigmentosa, Age-related macular degeneration	Wahlin et al., 2017 [48]; Llonch et al., 2018 [49]; DiStefano et al., 2018 [50]

## Abbreviations

2D	two-dimensional
3D	three-dimensional
hESCs	human embryonic stem cells
hiPSCs	human induced pluripotent stem cells
hPSCs	human pluripotent stem cells
CRISPR	clustered regularly interspaced short palindromic repeat
MSCs	mesenchymal stem cells
ECM	extracellular matrix
VSMCs	vascular smooth muscle cells
NSCs	neural stem cells
ZIKV	Zika virus
TLRs	Toll-like receptors
ASPM	abnormal spindle-like primary microcephaly
SVZ	sub-ventricular zone
IFL	inner fiber layer
TTX	tetrodotoxin
HP	Hippeastrine hydrobromide
AQ	Amodiaquine dihydrochloride dihydrate
hNPCs	human pluripotent stem cell-derived cortical neural progenitors cells
iHIOs	induced human intestinal organoids
DE	definitive endoderm
PCOs	prostate cancer organoids
CRPC	castrate-resistant prostate cancer
FTE	fallopian tube epithelium
FTO	fallopian tube organoid
HGSOC	High-grade serious ovarian cancer
FTSECs	fallopian tube secretory epithelial cells
ASD	autism spectrum disorder
CF	cystic fibrosis
CFTR	cystic fibrosis Transmembrane-conductor regulator
HO	hepatic organoids
ALG	Alagille syndrome
SCID	severe combined immunodeficiency
